# High levels of HtrA4 observed in preeclamptic circulation drastically alter endothelial gene expression and induce inflammation in human umbilical vein endothelial cells

**DOI:** 10.1016/j.placenta.2016.09.003

**Published:** 2016-11

**Authors:** Yao Wang, Guiying Nie

**Affiliations:** a Implantation and Placental Development Laboratory, Centre for Reproductive Health, Hudson Institute of Medical Research, Clayton, Victoria, 3168, Australia; b Department of Molecular and Translational Sciences, Monash University, Clayton, Victoria, 3800, Australia; c Department of Biochemistry and Molecular Biology, Monash University, Clayton, Victoria, 3800, Australia

**Keywords:** Preeclampsia, Early-onset, Endothelial dysfunction, HtrA4, Inflammation

## Abstract

**Introduction:**

Preeclampsia (PE) is a life-threatening pregnancy disorder characterized by wide-spread endothelial dysfunction. Placental factors circulating in the maternal blood are believed to cause endothelial dysfunction. Our previous study identified HtrA4 as a placenta-specific serine protease that is released into the maternal circulation and significantly increased in early-onset PE. In this study, we examined the impact of HtrA4 on expression of endothelial genes related to vessel biology, using human umbilical vein endothelial cells (HUVECs) as a model.

**Methods:**

HUVECs were treated with 0 or 3 μg/ml HtrA4 (highest concentration seen in PE circulation) for 24 h and analysed by an endothelial cell biology PCR array containing 84 genes. HtrA4-induced changes were then validated by real-time RT-PCR and ELISA for time and dose dependency.

**Results:**

High levels of HtrA4 significantly altered the expression of a range of genes related to inflammation, vaso-activity, angiogenesis, cell adhesion, platelet activation and coagulation. In particular, pro-inflammatory genes *IL6*, *PTGS2 (COX2)* and *IL1B* were significantly increased by HtrA4. IL6 protein in HUVEC media was also drastically increased. *THBD*, an anticoagulant factor reported to be increased in PE, was significantly up-regulated by HtrA4. In contrast, *THBS1*, which is involved in many regulatory processes of endothelial cell biology, was severely down-regulated by HtrA4.

**Discussion:**

HtrA4 significantly increased the inflammatory responses of HUVECs, and altered their expression of a number of genes important for vessel biology. These data suggest that placenta-derived HtrA4 that is increased in PE circulation is a potential causal factor of endothelial dysfunction.

## Highlights

• HtrA4 altered expression of endothelial genes involved in vessel biology.
• HtrA4 significantly induced inflammation in endothelial cells.
• Placental-derived HtrA4 is a potential causal factor of endothelial dysfunction.

## 1 Introduction

Preeclampsia (PE) is a life-threatening disorder of human pregnancy affecting 2–8% of pregnancies worldwide [1], [2]. PE can progress rapidly leading to multi-organ failure and symptoms are closely linked to wide-spread endothelial dysfunction [3]. It is well accepted that in PE, the placenta releases abnormal amounts/types of factors into the maternal circulation and these circulating factors contribute to endothelial dysfunction and the maternal syndrome of PE [4]. Significantly elevated circulating factors in PE include cytokines, antiangiogenic factors, syncytiotrophoblast microparticles and activated leukocytes [5], [6], [7], [8]. Changes in many of these factors are believed to reflect an exacerbated maternal response to pregnancy, which itself is considered as a low grade systemic inflammation [9]. One study has shown that serum from preeclamptic women is cytotoxic to endothelial cells *in vitro*, and that clinical condition improves drastically after 24–48 h postpartum when the cytotoxic activity is dramatically reduced [10].

Endothelial injury in PE is evidenced by the appearance of morphological lesions, glomerular endotheliosis, and increased circulating levels of fibronectin, von Willebrand factor and cytokines, all of which can be secreted by endothelial cells as an inflammatory reaction [11], [12]. Endothelial cells can also alter their synthesis of vaso-relaxing/vasoconstrictors and pro/anticoagulants upon endothelial injury in PE [11], and the damage to the maternal vasculature can persist many years after PE. A study revealed vascular endothelial defects in women even three years after a PE pregnancy, and the data suggested that the impairment was more severe with recurrent PE [13]. PE-induced endothelial injury has long-term and harmful consequences, for instance, women who had PE have a higher risk of developing cardiovascular diseases many years postpartum [14].

PE can be classified into two distinct subtypes, early-onset which occurs before 34 weeks of gestation, and late-onset PE which occurs after 34 weeks [15]. Emerging evidence strongly suggests that the two PE subtypes have vastly different etiologies, and early-onset PE poses a far more significant maternal risk, with a 20-fold higher mortality rate than late-onset PE [16], [17], [18], [19]. The risk of cardiovascular disease is also much higher in women who have had early-onset than late-onset PE [20], [21], [22], suggesting that endothelial injury is more profound in early-onset than late-onset PE.

We have recently reported that high temperature requirement A4 (HtrA4) is a placenta-specific protease that is significantly increased in the circulation of early-onset PE [23]. HtrA4 belongs to a serine protease family that also includes HtrA1, HtrA2 and HtrA3. These proteins are known to function as ATP-independent protein quality control factors to regulate cellular processes such as proliferation, unfolded stress response, programmed cell death and aging [24]. All HtrAs contain a trypsin-like serine protease domain and are proven to have catalytic activities [25]. HtrA1, HtrA3 and HtrA4 share a similar domain structure and are secreted out of cells, whereas HtrA2 has a transmembrane domain and is localized in mitochondria [26]. HtrA4 is the newest member of the mammalian HtrA family, it is expressed only by the placenta and secreted into the maternal circulation [23]. In a normal pregnancy, HtrA4 serum levels increase progressively to around 24–25 weeks of gestation, then remain stable throughout the remainder of the pregnancy [23].

Our study showed that in early-onset PE, both the placental production and circulating levels of HtrA4 are significantly increased [23]. We further demonstrated that HtrA4 at high concentrations seen in early-onset PE disrupted the tube formation of human umbilical vein endothelial cell (HUVEC), disturbed cellular integrity and increased cellular permeability [23]. These results suggest high levels of circulating HtrA4 of placental origin may contribute to endothelial dysfunction and the development of early-onset PE.

In this study, using HUVEC as a model, we first examined the impact of HtrA4 on expression of endothelial genes involved in vessel cell biology by an array approach, then validated the data by real-time RT-PCR and ELISA.

## 2 Materials and methods

### 2.1 Cell culture

HUVECs (ATCC, Maryland, USA) were cultured at 37 °C in a humidified atmosphere of 5% CO_2_ in air, and maintained in DMEM (Thermo Fisher Scientific, VIC, Australia) supplemented with 1% antibiotics (Thermo Fisher Scientific), 2 mM l-glutamine (Sigma-Aldrich, Missouri, USA), 1 mM sodium pyruvate (Thermo Fisher Scientific) and 10% fetal bovine serum (Thermo Fisher Scientific). The starting passage of the HUVECs was 13, and the experiments were completed within eight passages. The cells were cultured in 12-well plates (Thermo Fisher Scientific) at 1 × 10^5^ per well density for 24 h (h), then treated with recombinant HtrA4 (BioTeZ, Berlin, Germany, 1.5 μg/ml or 3.0 μg/ml) or vehicle control for 24 or 48 h. The vehicle control contains 150 mM NaCl, 5 mM CaCl_2_, 50 mM Tris-HCl pH 7.5, 0.05% Brij 35 solution, 50 mM imidazole in ultrapure H_2_O. The two doses of HtrA4 were chosen to represent the median and highest levels of HtrA4 found in early-onset PE circulation [23]. After the treatment, media were collected and cells were used for RNA extraction. The experiment was repeated four times.

### 2.2 RNA extraction

RNA was extracted using RNeasy Mini Kit (Qiagen, Hilden, Germany) and contaminating DNA was removed using RNase-free DNase (Qiagen) according to the manufacturers' protocols. The RNA concentration was determined using Nanodrop ND-1000 (Thermo Fisher Scientific).

### 2.3 PCR array for endothelial cell biology

RNA samples from vehicle control or 3 μg/ml HtrA4 treatment for 24 h were pooled from three independent experiments, and 500 ng of the pooled RNA presenting control and HtrA4 treatment (3 μg/ml, 24 h) was reverse transcribed into complementary DNA (cDNA) using RT^2^ First Strand Kit (Qiagen). A RT^2^ Profiler 84 gene PCR array (Qiagen) was screened as per manufacturer's instruction on an ABI 7900 HT Fast real-time machine (Applied Biosystems, VIC, Australia). The results were analysed using Qiagen RT^2^ profiler PCR array data analysis software.

### 2.4 Real-time RT-PCR analysis

Genes showing more than 2-fold differences in expression on the array between the vehicle control and HtrA4 treatment were validated by real-time RT-PCR. RNA (300 ng) from three independent experiments of 24 h and 48 h treatment with 0, 1.5 μg/ml and 3.0 μg/ml HtrA4 was reverse transcribed in 20 μl using SuperScript III First-Strand kit (Invitrogen, VIC, Australia) as per manufacturer's protocol. Real-time RT-PCR was performed with primers specified in Supplementary Table 1 on an ABI 7900 HT Fast real-time machine with the following conditions: 1) 95 °C for 10 min for enzyme activation, 2) 40 cycles of denaturation (15 s at 95 °C), annealing (5 s at 58 °C), extension (10 s at 72 °C), and a single fluorescence measurement at 70–75 °C for quantitation, and 3) dissociation curve assessment between 60 °C and 95 °C with continuous fluorescence measurement. All cDNA samples were diluted 1:80, and PCR reaction was prepared to a final volume of 10 μl with 5 μl SYBR Green PCR Master Mix (Applied Biosystems), 4 μl diluted cDNA sample and 0.5 μM final concentration of forward and reverse primers. All samples were run in triplicates, each gene expression were normalised to 18 S, and fold changes were calculated using ΔΔCt.

### 2.5 Enzyme-linked immunosorbent assay (ELISA)

The secreted levels of interleukin (IL)6 and monocyte chemoattractant protein (MCP)1 in the above treated cell media were measured by ELISA (Ray Biotech, Georgia, USA) according to manufacturer's instruction.

### 2.6 Cytokine antibody array

A cytokine antibody array for 36 cytokines (R&D System, Minnesota, USA) was analysed to determine whether HtrA4 alters these cytokines in HUVECs. Conditioned media from cells treated with vehicle control or 3.0 μg/ml HtrA4 for 24 h from four independent experiments were pooled for the array analysis. Dots representing 36 cytokines were analysed by densitometry using ImageJ software (National Institutes of Health, USA).

### 2.7 Statistical analysis

Statistical analyses were conducted using GraphPad Prism (v.6, GraphPad Software Inc., CA). Data are expressed as mean ± SD, comparison was made using two-way ANOVA followed by Tukey's post-hoc tests, and difference was considered significant if p < 0.05.

## 3 Results

We used HUVECs as a model and examined the effect of HtrA4 on the expression of 84 genes that are involved in endothelial cell biology (Supplementary Table 2). These genes were broadly categorised into four groups: inflammation, angiogenesis and vaso-activities, platelet activation and cell adhesion, coagulation and apoptosis. Total RNA from cells treated with vehicle control or 3 μg/ml HtrA4 (highest level detected in early-onset PE serum) for 24 h was analysed on the array. Genes that showed more than 2-fold changes in expression were then validated by real-time RT-PCR for time and dose dependency, using cells treated with vehicle control, 1.5 μg/ml or 3 μg/ml HtrA4 for 0, 24 or 48 h. The array data are presented together with the real-time RT-PCR validation for each of the four gene groups categorised above.

### 3.1 HtrA4 regulation of genes involved in endothelial inflammation

**Fig. 1:**
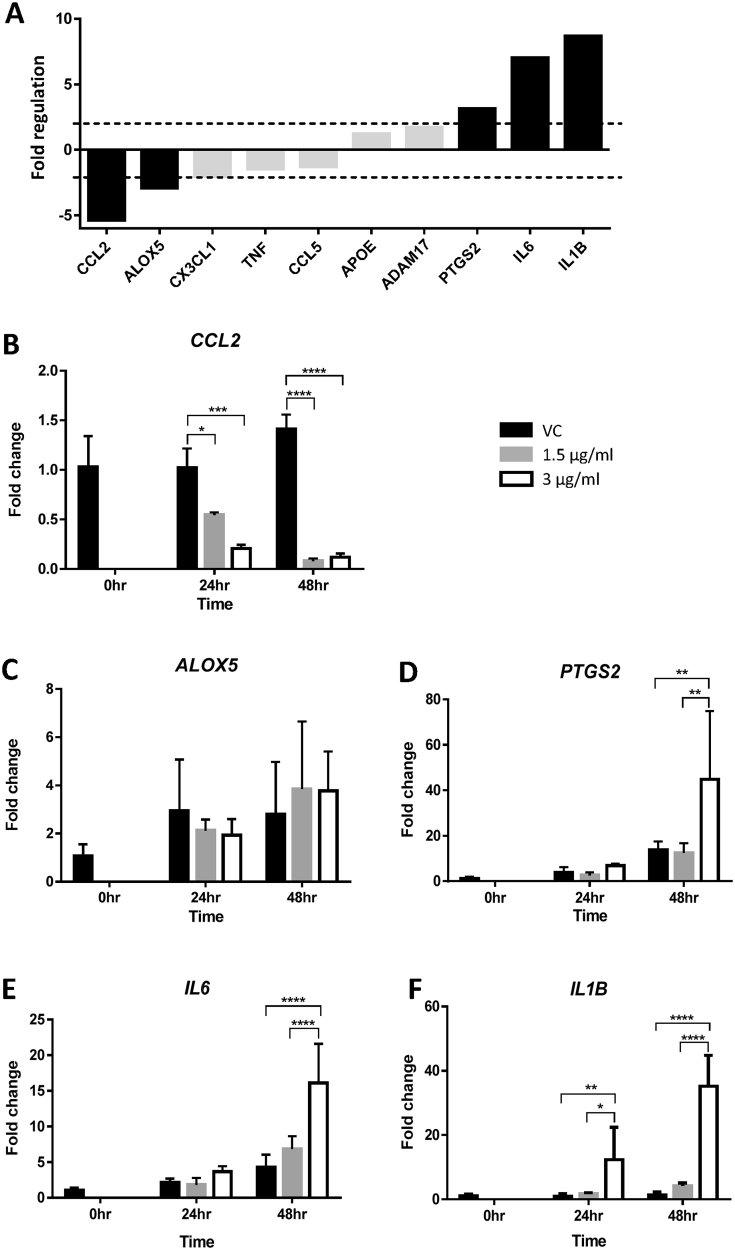
HtrA4-induced changes in mRNA expression of genes involved in inflammatory responses. A) PCR array data. RNA samples from vehicle control (vc) or 3 μg/ml HtrA4 treatment for 24 h were pooled from 3 experiments for the array. Data is expressed as HtrA4-induced fold changes relative to the control. Genes that showed >2-fold differences (in black bar) were chosen for validation by real-time RT-PCR. (B–F) Real-time RT-PCR analysis of *CCL2* (B), *ALOX5* (C), *PTGS2* (D), *IL6* (E) and *IL1B* (F). Cells were treated with 0, 1.5 μg/ml or 3 μg/ml HtrA4 for 0, 24 or 48 h, n = 3. Data is expressed as mean ± SD. *p < 0.05, **p < 0.01, ***p < 0.001, ****p < 0.0001.

Among the ten genes related to inflammation, five were altered by HtrA4 by more than 2 folds on the PCR array, two down-regulated and three up-regulated (Fig. 1A). These five genes were further examined by real-time RT-PCR for time and HtrA4 dose dependency. For the two down-regulated genes, *CCL2* (also known as *MCP1*) and *ALOX5*, *CCL2* mRNA was significantly reduced by HtrA4 even at the lower dosage of 1.5 μg/ml; and the reduction was more profound at 48 h than 24 h (Fig. 1B). Although *ALOX5* showed a 2.9-fold reduction on the array, real-time RT-PCR did not find significant changes in *ALOX5* mRNA across all treatment conditions (Fig. 1C). This is likely due to the low expression of *ALOX5* in HUVECs. For the three up-regulated genes (Fig. 1A), *PTGS2* (also called *COX2*), *IL6* and *IL1B* were all validated by real-time RT-PCR. *PTGS2* mRNA was significantly up-regulated by 3 μg/ml HtrA4 at 48 h time point (Fig. 1D). *IL6* mRNA was significantly increased by 3 μg/ml HtrA4 at the longer time point (Fig. 1E). In contrast, *IL1B* expression was increased by HtrA4 dose- and time-dependently (Fig. 1F).

### 3.2 HtrA4 regulation of genes associated with angiogenesis and vaso-activities

**Fig. 2:**
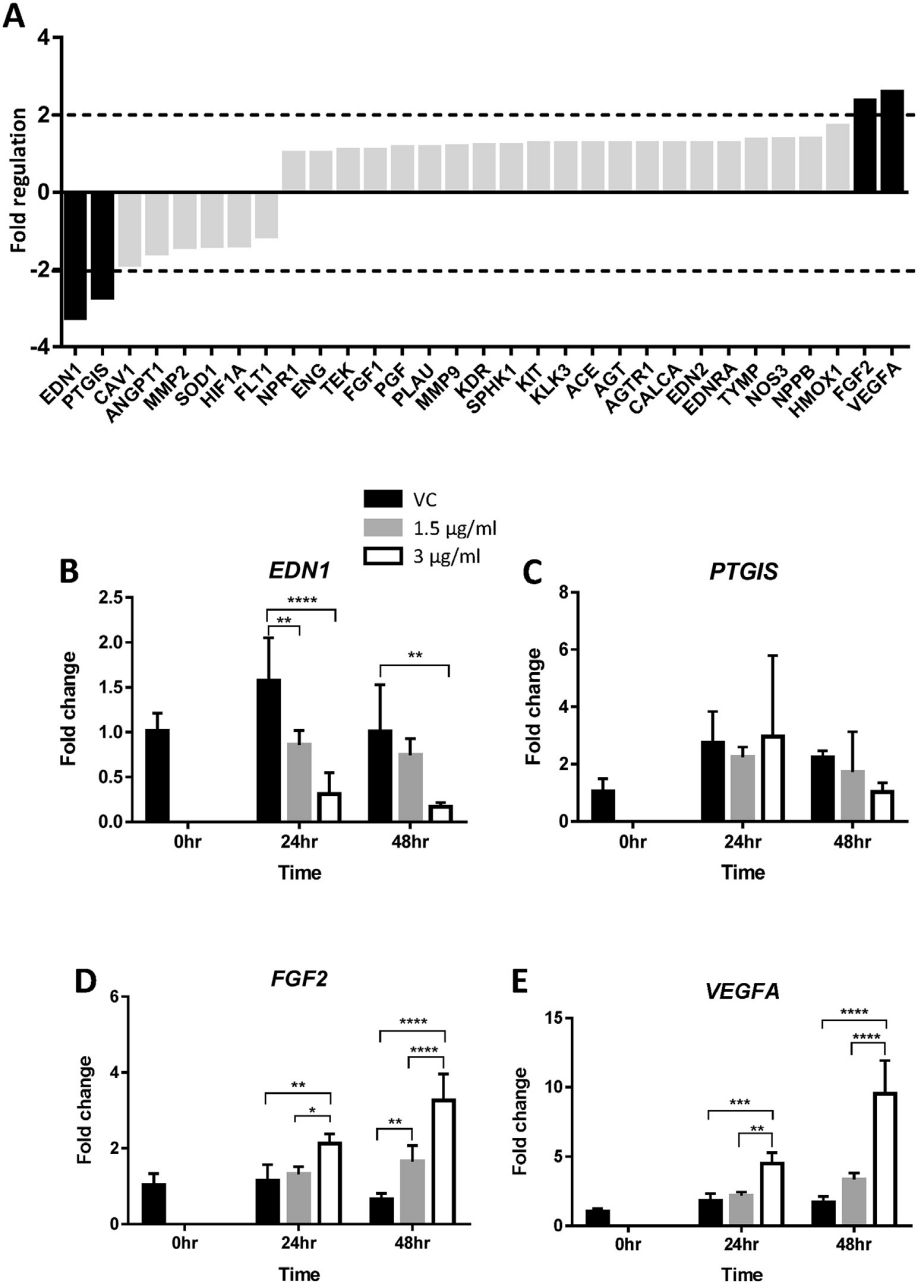
HtrA4-induced changes in mRNA expression of genes involved in vaso-activities and angiogenesis. A) PCR array data. RNA samples from vehicle control (vc) or 3 μg/ml HtrA4 treatment for 24 h were pooled from 3 experiments for the array. Data is expressed as HtrA4-induced fold changes relative to the control. Genes showed >2-fold differences in mRNA (in black bar) were chosen for validation by real-time RT-PCR. (B–E) Real-time RT-PCR analysis of *EDN1* (B), *PTGIS* (C), *FGF2* (D) and *VEGFA* (E). Cells were treated with 0, 1.5 μg/ml or 3 μg/ml HtrA4 for 0, 24 or 48 h, n = 3. Data is expressed as mean ± SD. *p < 0.05, **p < 0.01, ***p < 0.001, ****p < 0.0001.

Thirty-one genes on the array were categorised into this group (Fig. 2A). The PCR array analysis identified two down-regulated (*EDN1* and *PTGIS*) and two up-regulated (*FGF2* and *VEGFA*) genes (Fig. 2A). Real-time RT-PCR confirmed that *EDN1* was significantly down-regulated by HtrA4 in a dose- and time-dependent manner (Fig. 2B), but *PTGIS* mRNA was not altered by HtrA4 (Fig. 2C). The two up-regulated genes, *FGF2* and *VEGFA*, were confirmed by real-time PCR to be significantly increased by HtrA4 (Fig. 2D–E). The up-regulation was greatest at 48 h with 3 μg/ml HtrA4 treatment (Fig. 2D–E).

### 3.3 HtrA4 regulation of cell adhesion and platelet activation genes

**Fig. 3:**
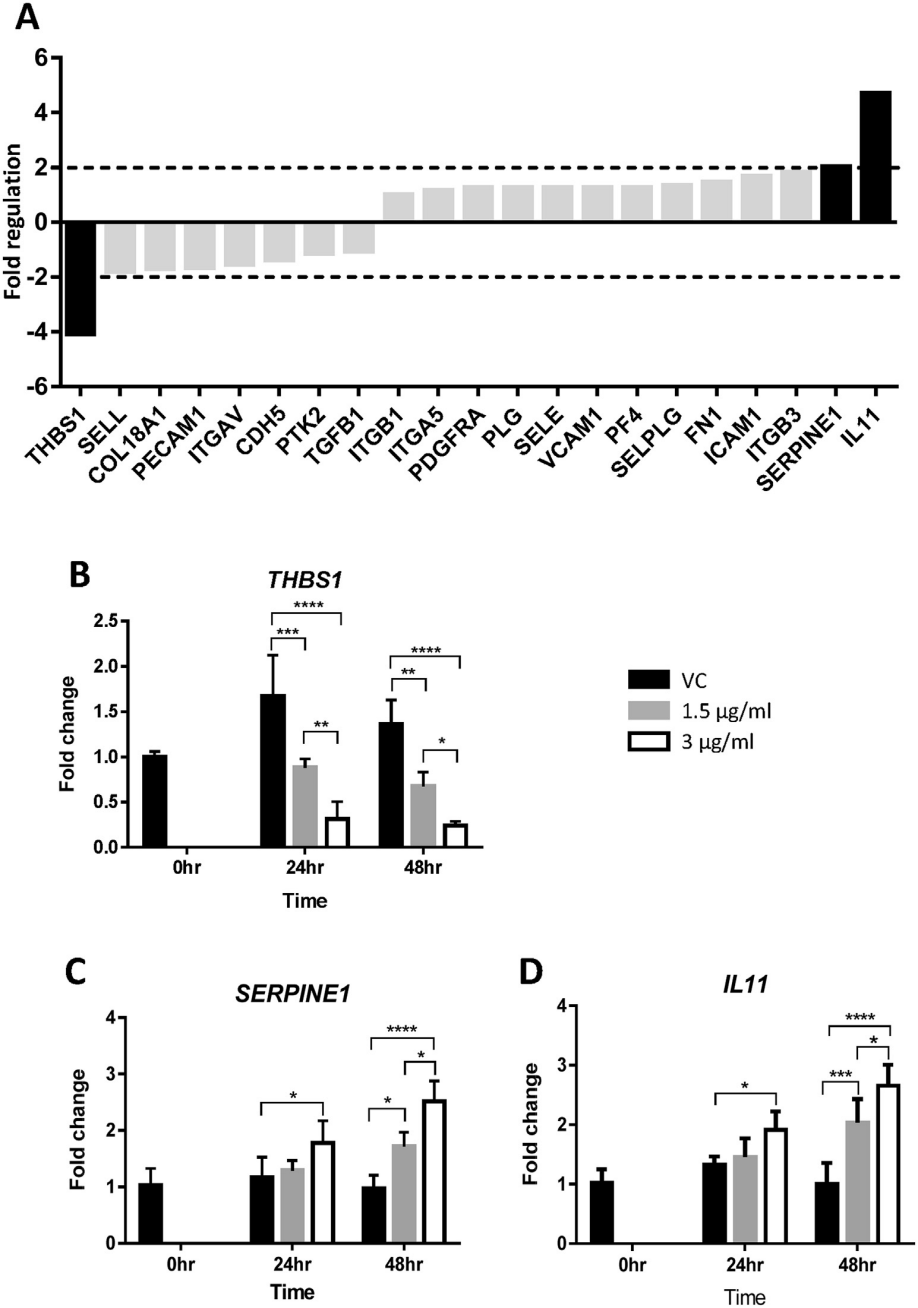
HtrA4-induced changes in mRNA expression of genes involved in cell adhesion and platelet activation. A) PCR array data. RNA samples from vehicle control (vc) or 3 μg/ml HtrA4 treatment for 24 h were pooled from 3 experiments for the array. Data is expressed as HtrA4-induced fold changes relative to the control. Genes showed >2-fold differences in mRNA (in black bar) were chosen for validation by real-time RT-PCR. (B–D) Real-time RT-PCR analysis of *THBS1* (B), *SERPINE1* (C) and *IL11* (D). Cells were treated with 0, 1.5 μg/ml or 3 μg/ml HtrA4 for 0, 24 or 48 h, n = 3. Data is expressed as mean ± SD. *p < 0.05, **p < 0.01, ***p < 0.001, ****p < 0.0001.

Twenty-two genes on the array were in this category (Fig. 3A). The PCR array identified one down-regulated (*THBS1*) and two up-regulated (*SERPINE1* and *IL11*) genes (Fig. 3A). *THBS1* is responsible for cell adhesion, whereas *SERPINE1* and *IL11* are involved in platelet activation. Real-time RT-PCR confirmed that *THBS1* mRNA was significantly down-regulated by HtrA4 in a dose- and time-dependent manner (Fig. 3B). Both *SERPINE1* and *IL11* were also validated by rea-time RT-PCR to be significantly up-regulated by HtrA4 dose- and time-dependently (Fig. 3C–D).

### 3.4 HtrA4 regulation of genes involved in apoptosis and coagulation

**Fig. 4:**
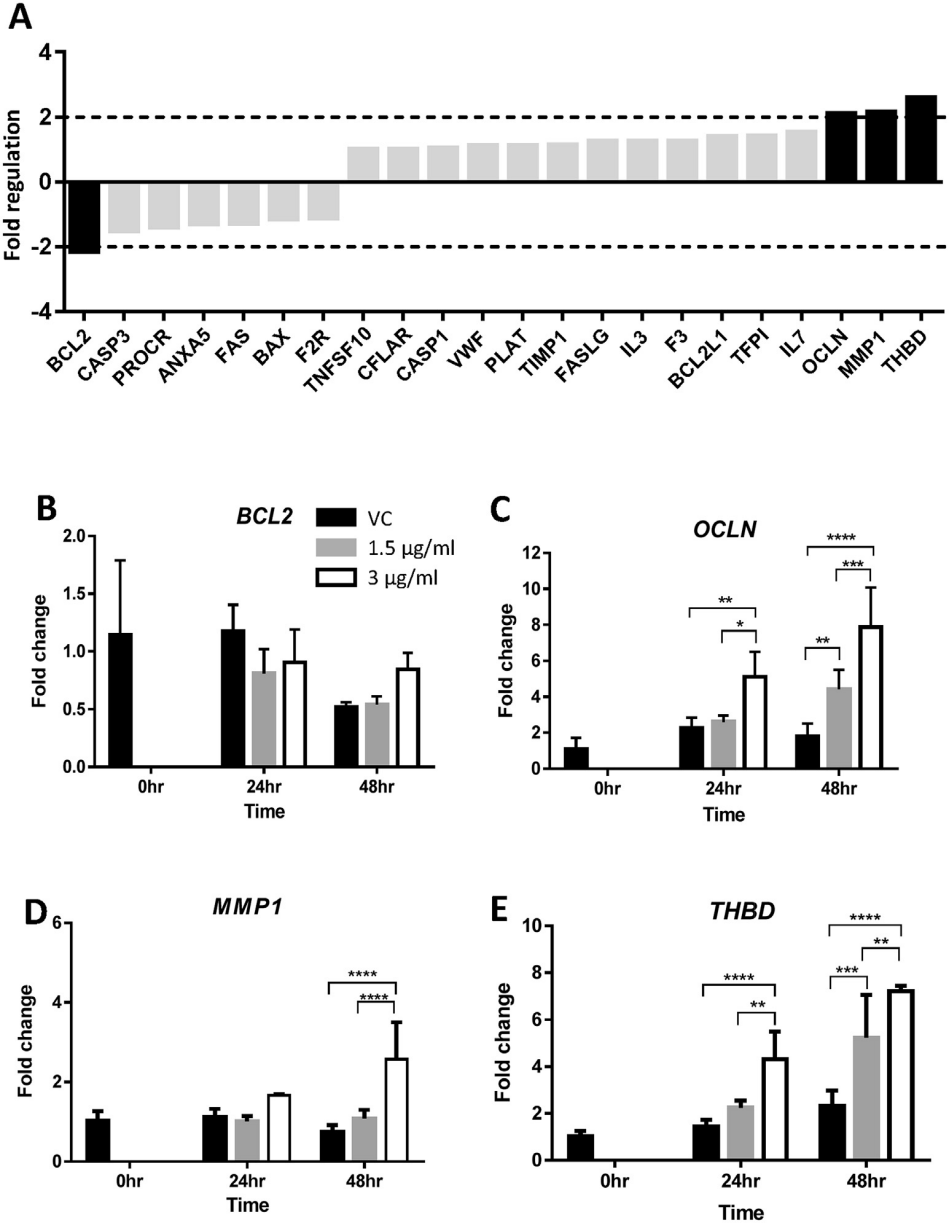
HtrA4-induced changes in mRNA expression of genes involved in apoptosis and coagulation. A) PCR array data. RNA samples from vehicle control (vc) or 3 μg/ml HtrA4 treatment for 24 h were pooled from 3 experiments for the array. Data is expressed as HtrA4-induced fold changes relative to the control. Genes showed >2-fold differences in mRNA (in black bar) were chose for validation by real-time RT-PCR. (B–E) Real-time RT-PCR analysis of *BCL2* (B), *OCLN* (C), *MMP1* (D) and *THBD* (E). Cells were treated with 0, 1.5 μg/ml or 3 μg/ml HtrA4 for 0, 24 or 48 h, n = 3. Data is expressed as mean ± SD. *p < 0.05, **p < 0.01, ***p < 0.001, ****p < 0.0001. Data shown as mean ± SD.

There were twenty-two genes in this group (Fig. 4A). The PCR array identified one down-regulated (BCL2) and three up-regulated (*OCLN*, *MMP1* and *THBD*) genes (Fig. 4A). *BCL2*, which is essential for apoptosis, showed no HtrA4-induced changes in mRNA expression by real-time RT-PCR (Fig. 4B). For the three up-regulated genes, *OCLN*, encoding a cell-junction protein, was confirmed by real-time RT-PCR to be up-regulated by HtrA4, and most prominently at 48 h (Fig. 4C). For the two up-regulated genes that are involved in coagulation, *MMP1* mRNA was significantly increased by 3 μg/ml HtrA4 at 48 h (Fig. 4D). In contrast, *THBD* mRNA was significantly increased by HtrA4 in a dose- and time-dependent manner (Fig. 4E).

In total, thirteen genes were confirmed by real-time RT-PCR to be significantly altered by HtrA4, ten were up-regulated and three down-regulated (Table 1). Functions of these thirteen genes and their potential relevance to endothelial dysfunction and PE are also summarised in Table 1.

### 3.5 Validation of HtrA4 regulation of pro-inflammatory factors at the protein level

**Fig. 5:**
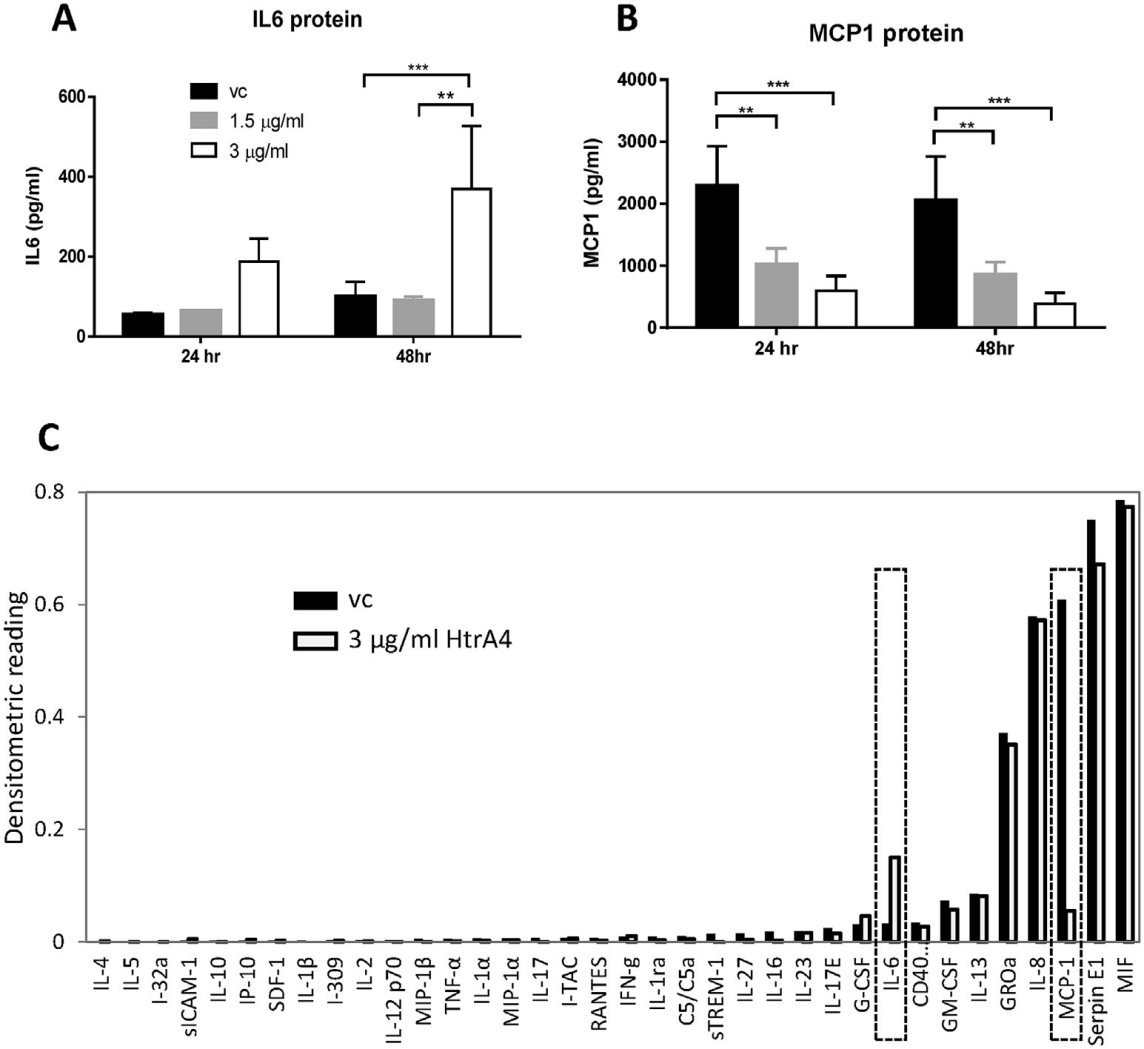
HtrA4-induced changes in cytokines at the protein level. A-B) ELISA detection of IL6 (A) and MCP1 (B) in media of HUVECs following treatment with 0, 1.5 μg/ml or 3 μg/ml HtrA4 for 24 or 48 h, n = 4. Data is expressed as mean ± SD. *p < 0.05, **p < 0.01, ***p < 0.001. C) Analysis of 36 cytokines by an antibody array. Media from HUVECs treated with vehicle control (vc) or 3 μg/ml HtrA4 for 24 h were pooled from 4 independent experiments and analysed on the array. The graph shows densitometric reading of each cytokine. Significant changes were detected only for IL6 and MCP1, consistent with the ELISA data shown in (A–B).

As heightened inflammation is a key feature of endothelial dysfunction and PE, we selected two genes from the inflammation group, one up-regulated (*IL6*, Fig. 1F) and one down-regulated (*CCL2*, Fig. 1B) for validation at the protein level to further confirm the real-time RT-PCR data. The levels of these two cytokines in HUVEC media were measured by ELISA. IL6 protein was significantly increased by 3 μg/ml HtrA4 at 48 h time points, the lower dose of HtrA4 (1.5 μg/ml) and the shorter time point (24 h) had no significant effect on IL6 protein (Fig. 5A). This is highly consistent with the real-time RT-PCR data (Fig. 1E). On the other end of the spectrum, the only down-regulated inflammatory gene, *CCL2*, which encodes MCP1, was significantly reduced at the protein level by HtrA4 in a dose- and time-dependent manner (Fig. 5B), and the pattern is identical to its mRNA changes (Fig. 1B).

As both IL6 and MCP1 are cytokines and only a few cytokines were on the PCR array, we further examined whether HtrA4 affects a broad range of cytokines using an antibody array for 36 cytokines (Fig. 5C). HUVEC media treated with either vehicle control or 3 μg/ml HtrA4 for 24 h were pooled from four independent experiments and applied onto the cytokine array. Of the thirty-six cytokines examined, IL6 showed a 4.6-fold increase and MCP1 displayed a 11-fold reduction by HtrA4 compared to vehicle control (Fig. 5C), the other thirty-four cytokines examined showed no significant difference between vehicle control and HtrA4 treatment (Fig. 5C). These data further confirmed the specific and opposing effect of HtrA4 on IL6 and MCP1.

## 4 Discussion

This study demonstrated that HtrA4 significantly affected endothelial cell gene expression. At a concentration found in early-onset PE circulation, HtrA4 profoundly altered a range of endothelial genes related to inflammation, vaso-activity, angiogenesis, cell adhesion, platelet activation and coagulation. HtrA4 also significantly increased HUVEC release of pro-inflammatory cytokine IL6.

Several studies reported that maternal serum IL6 is significantly increased in PE patients [27]. One study in particular demonstrated that serum IL6 was much higher in women with early-onset PE compared to late-onset PE or normotensive controls [28]. The same study also showed that placental IL6 expression was much lower in early-onset PE, and suggested that the increase in serum IL6 was more likely resulted from maternal endothelial dysfunction than a direct consequences of defective placenta [28]. Our current study provides strong support for this view. Furthermore, HtrA4 also significantly up-regulated two other major pro-inflammatory genes, *PTGS2* (*COX2*) and *IL1B*, further suggesting that the circulating HtrA4 of placenta-origin can intensify the inflammatory response of maternal endothelial cells, which is frequently observed in early-onset PE [29], [30], [31].

Additional inflammatory factors such as TNF-α are reported to be significantly elevated in preeclamptic serum [9], [32], it is likely that many of these factors are released directly by the placenta. Endothelial cells are always considered to be the major site of inflammatory response in PE, this study supports this view and suggests that maternal endothelial cells also release multiple inflammatory factors to the circulation as a response to placental factors such as HtrA4.

The only down-regulated gene identified in the inflammation category was *CCL2*, which encodes for MCP-1. The main function of this protein is to recruit monocytes or macrophages to the site of inflammation as a result of tissue injury or infection [33], [34]. MCP1 is involved in many human diseases, and is expressed in many cell types including the endothelial cells [33]. MCP1 is reported to be higher in PE women [35], however, the majority of these reports relate to placental production of MCP1, which acts locally to recruit macrophages from the blood stream across the endothelium [36], [37]. In particular, MCP1 secretion is reported to be elevated in placental mesenchymal stromal cells isolated from PE patients [38]. However, the regulation of MCP1 expression in endothelial cells and its role in PE has not been investigated. Our data showed a significant reduction of endothelial MCP1 (both mRNA and protein) by HtrA4, this may have important implications in the effectiveness of endothelial cells to recruit macrophages.

Our data also showed that HtrA4 affected several regulatory factors of endothelial cell biology. For Instance, *THBD*, an endothelial cell-specific membrane-bound receptor that functions as an anticoagulant, was highly up-regulated by HtrA4. This is consistent with other studies showing that serum THBD is increased in PE women compared to normal controls [39], [40], and that THBD is a potential marker of endothelial dysfunction to predict PE [39]. Furthermore, increased levels of THBD in the maternal circulation negatively correlate to infant birth weight, and the highest plasma THBD level was detected in PE pregnancy with intrauterine growth restriction, which is often associated with early-onset PE. It was suggested that the damage to endothelium as a result of THBD dysregulation could have a major impact on fetal development [41].

*SERPINE1*, another gene up-regulated by HtrA4, encodes the inhibitor for fibrinolysis and is also elevated in the circulation of PE women [42]. Dysregulation of SERPINE1 protein is reported to inhibit fibrin degradation, leading to fibrosis and formation of blood clot in the blood vessel [43]. *THBS1*, which is reported to be lower in women with severe PE [44], was significantly down-regulated by HtrA4. THBS1 is involved in many regulatory processes of endothelial cell function such as adhesion, motility and proliferation, and *THBS1* down-regulation can significantly impair the normal cellular function of endothelial cells [44].

Surprisingly, only a small number of genes in the angiogenic and vasoactive group were found to be regulated by HtrA4. This suggest that endothelial genes in this group may not be the major targets of HtrA4 action, and other placental-derived factors may be responsible for their dysregulation and contribute to the endothelial dysfunction.

Overall, our study suggests that high levels of placental-derived HtrA4 that is circulating in early-onset PE women is a potential causal factor of endothelial dysfunction. HtrA4 profoundly altered HUVEC expression of several factors essential for normal endothelial cell function and inflammation responses. As endothelial dysfunction is a major aspect of early-onset PE development, our study provides new insight into the underlying causes of this disease.

One limitation of this study is that HUVEC was used as a model. HUVEC is a well characterized cell line and commonly used for studying endothelial biology, but it may not reflect all the features of human vascular endothelial cells. Future studies will confirm the impact of HtrA4 on endothelial cells using other cell models.

## Conflict of interests

The authors declare no conflict of interests.

## Figures and Tables

**Table 1:** List of genes that were validated by real-time RT-PCR to be significantly affected by HtrA4.

Gene category	Gene name	Regulation by HtrA4	Functions in endothelial cells
Inflammatory Response	*CCL2/MCP1*		Involved in immnoregulatory and inflammatory processes, recruitment of monocytes and macrophages
	*PTGS2/COX2*		A key enzyme in prostaglandin biosynthesis, and is involved in inflammation and mitogenesis
	*IL6*		Major functions in inflammation and the maturation of B cells, highly up-regulated in PE circulation
	*IL1B*		Mediates inflammatory response and various cellular activities, including proliferation and differentiation
Angiogenesis and Vaso-activities	*EDN1/ET1*		A secreted peptide that acts as a vasoconstrictor
	*FGF2*		Involves in ranges of biological processes, including mitogenic and angiogenic activities
	*VEGFA*		Acts specifically on endothelial cells and has various functions, including angiogenesis and cell growth
Platelet Activation and Cell Adhesion	*THBS1*		Mediates cell-to-cell and cell-to-matrix interactions and plays a role in platelet aggregation and angiogenesis
	*SERPINE1*		A major inhibitor of tissue plasminogen activator and urokinase, acts on blood vessels to inhibit fibrinolysis
	*IL11*		Stimulates the proliferation of hematopoietic cells and megakaryocyte to increase platelet production
Coagulation and Apoptosis	*OCLN*		An integral membrane protein that is required for cytokine-induced regulation of the tight junction
	*MMP1*		Involves in the breakdown of extracellular matrix in many physiological processes
	*THBD*		An endothelial-specific receptor that binds to thrombin to activate protein C, which degrades clotting factors. It is a marker for endothelial dysfunction
