# Supplementary material for: High levels of HtrA4 observed in preeclamptic circulation drastically alter endothelial gene expression and induce inflammation in human umbilical vein endothelial cells
Source: Placenta. 2016 Nov;47:46–55. doi: 10.1016/j.placenta.2016.09.003 (PMC5090051; doi:10.1016/j.placenta.2016.09.003)
Supplement: Supplementary file 1 [file mmc1.docx]

**Supplementary table 1.** Primer sequences for real-time RT-PCR

| Gene symbol | Accession Number | Primer sequence (5'→3') | Product  Size (bp) |
| --- | --- | --- | --- |
| *ALOX5* | NM_000698 | Forward CTACATCTACCTCAGCCTCG  Reverse CCAGTCGTCATTCAGCCAGT | 184 |
| *BCL2* | NM_000633 | Forward ACGCCCCATCCAGCCGCAT  Reverse TCACCCCGTCCCTGAAGAGC | 262 |
| *CCL2* | NM_002982 | Forward AGTCTCTGCCGCCCTTCTGT  Reverse ATCCTGAACCCACTTCTGCT | 250 |
| *EDN1* | NM_001955 | Forward CCTTCCTCCATCCCCCATAC  Reverse CCAACCTCTTTCATTAGCCG | 266 |
| *FGF2* | NM_002006 | Forward CGGATGGGGGTAGTGAGCA  Reverse ATCTTGAGGTGGAAGGGTCT | 180 |
| *IL1B* | NM_000576 | Forward GGGCTGGCAGAAAGGGAACA  Reverse GGGAGCGAATGACAGAGGGT | 270 |
| *IL6* | NM_000600 | Forward CCCCTGACCCAACCACAAAT  Reverse ACAACAATCTGAGGTGCCCA | 165 |
| *IL11* | NM_000641 | Forward CCCAAAGCCACCACCGTCCT  Reverse ACCCCAGTCCCCTCCTCCTC | 227 |
| *MMP1* | NM_002421 | Forward CAGGTATTGGAGGGGATGCT  Reverse ACGCTTTTGGGGTTTGTGGG | 267 |
| *OCLN* | NM_002538 | Forward CTCTCTCAGCCAGCCTACTC  Reverse GTTCCATAGCCTCTGTCCCA | 170 |
| *SERPINE1* | NM_000602 | Forward CCTGCTTCCACCCGTCTCTC  Reverse ACATTCACTCTGCCACCTGC | 283 |
| *THBD* | NM_000361 | Forward CCTGTGCCTCCTCACCCCCA  Reverse CCAATAACGCTCACCCTCCT | 266 |
| *THBS1* | NM_003246 | Forward GAACAGGAAGAAGCGTAAAGAC  Reverse TGAGCACAAGGGGCAGAGCA | 172 |
| *PTGIS* | NM_000961 | Forward ACTCCCCCCTTCCAAATCAG  Reverse CTAACCCACTCATCTCTCCC | 287 |
| *PTGS2* | NM_000963 | Forward CAGTCTTCTCATCACTTCGT  Reverse TTCCAGTCACAAACCCCGTA | 316 |
| *VEGFA* | NM_001171623 | Forward GGAGGGGGAGGAGGAAGAAG  Reverse GGAGGTAGAGCAGCAAGGCA | 317 |

**Supplementary table 2.** Genes on the endothelial cell biology PCR array

| Gene category | Gene name | Full name |
| --- | --- | --- |
| Inflammatory Response | *ADAM17* | ADAM Metallopeptidase Domain 17 |
|  | *ALOX5* | Arachidonate 5-Lipoxygenase |
|  | *APOE* | Apolipoprotein E |
|  | *CCL2/MCP1* | Chemokine (C-C Motif) Ligand 2/ Monocyte Chemoattractant Protein-1 |
|  | *CCL5* | Chemokine (C-C Motif) Ligand 5 |
|  | *CX3CL1* | Chemokine (C-X3-C Motif) Ligand 1 |
|  | *IL6* | Interleukin 6 |
|  | *IL1B* | Interleukin 1, Beta |
|  | *PTGS2/COX2* | Prostaglandin-Endoperoxide Synthase 2/ Cyclooxygenase-2 |
|  | *TNF* | Tumor Necrosis Factor |
| Angiogenesis and Vaso-activities | *ACE* | Angiotensin I Converting Enzyme |
|  | *AGT/SERPINA8* | Angiotensinogen/Serpin Peptidase Inhibitor, Clade A, Member 8 |
|  | *AGTR1* | Angiotensin II Receptor, Type 1 |
|  | *ANGPT1* | Angiopoietin 1 |
|  | *CALCA* | Calcitonin-Related Polypeptide Alpha |
|  | *CAV1* | Caveolin 1 |
|  | *EDN1* | Endothelin 1 |
|  | *EDN2* | Endothelin 2 |
|  | *EDNRA* | Endothelin Receptor Type A |
|  | *ENG* | Endoglin |
|  | *FGF1* | Fibroblast Growth Factor 1 (Acidic) |
|  | *FGF2* | Fibroblast Growth Factor 2 (Basic) |
|  | *FLT1* | Fms-Related Tyrosine Kinase 1 |
|  | *HIF1A* | Hypoxia Inducible Factor 1, Alpha Subunit |
|  | *HMOX1* | Heme Oxygenase 1 |
|  | *KDR/* FLK1 | Kinase Insert Domain Receptor/Fetal Liver Kinase-1 |
|  | *KIT* | V-Kit Hardy-Zuckerman 4 Feline Sarcoma Viral Oncogene Homolog |
|  | *KLK3* | Kallikrein-Related Peptidase 3 |
|  | *MMP2* | Matrix Metallopeptidase 2 |
|  | *MMP9* | Matrix Metallopeptidase 9 |
|  | *NOS3* | Nitric Oxide Synthase 3 |
|  | *NPPB* | Natriuretic Peptide B |
|  | *NPR1* | Natriuretic Peptide Receptor 1 |
|  | *PGF* | Placental Growth Factor |
|  | *PLAU* | Plasminogen Activator, Urokinase |
|  | *PTGIS* | Prostaglandin I2 (Prostacyclin) Synthase |
|  | *SOD1* | Superoxide Dismutase 1, Soluble |
|  | *SPHK1* | Sphingosine Kinase 1 |
|  | *TEK* | TEK Tyrosine Kinase, Endothelial |
|  | *TYMP* | Thymidine Phosphorylase |
|  | *VEGFA* | Vascular Endothelial Growth Factor A |
| Platelet Activation and Cell Adhesion | *CDH5* | Cadherin 5, Type 2 (Vascular Endothelium) |
|  | *COL18A1* | Collagen, Type XVIII, Alpha 1 |
|  | *FN1* | Fibronectin 1 |
|  | *ICAM1* | Intercellular Adhesion Molecule 1 |
|  | *IL11* | Interleukin 11 |
|  | *ITGA5* | Integrin, Alpha 5 |
|  | *ITGAV* | Integrin, Alpha V |
|  | *ITGB1* | Integrin, Beta 1 |
|  | *ITGB3* | Integrin, Beta 3 |
|  | *PDGFRA* | Platelet-Derived Growth Factor Receptor, Alpha Polypeptide |
|  | *PECAM1* | Platelet/Endothelial Cell Adhesion Molecule 1 |
|  | *PF4* | Platelet Factor 4 |
|  | *PLG* | Plasminogen |
|  | *PTK2* | Protein Tyrosine Kinase 2 |
|  | *SELE* | Selectin E |
|  | *SELL* | Selectin L |
|  | *SELPLG* | Selectin P Ligand |
|  | *SERPINE1* | Serpin Peptidase Inhibitor, Clade E, Member 1 |
|  | *TGFB1* | Transforming Growth Factor, Beta 1 |
|  | *THBS1* | Thrombospondin 1 |
|  | *VCAM1* | Vascular Cell Adhesion Molecule 1 |
| Coagulation and Apoptosis | *ANXA5* | Annexin A5 |
|  | *BAX* | BCL2-Associated X Protein |
|  | *BCL2* | B-Cell CLL/Lymphoma 2 |
|  | *BCL2L1* | BCL2-Like 1 |
|  | *CASP1* | Caspase 1, Apoptosis-Related Cysteine Peptidase |
|  | *CASP3* | Caspase 3, Apoptosis-Related Cysteine Peptidase |
|  | *CFLAR* | CASP8 and FADD-Like Apoptosis Regulator |
|  | *F2R* | Coagulation Factor II (Thrombin) Receptor |
|  | *F3* | Coagulation Factor III |
|  | *FAS* | Fas Cell Surface Death Receptor |
|  | *FASLG* | Fas Ligand (TNF Superfamily, Member 6) |
|  | *IL3* | Interleukin 3 |
|  | *IL7* | Interleukin 7 |
|  | *MMP1* | Matrix Metallopeptidase 1 |
|  | *OCLN* | Occludin |
|  | *PLAT* | Plasminogen Activator, Tissue |
|  | *PROCR* | Protein C Receptor, Endothelial |
|  | *TFPI* | Tissue Factor Pathway Inhibitor |
|  | *THBD* | Thrombomodulin |
|  | *TIMP1* | TIMP Metallopeptidase Inhibitor 1 |
|  | *TNFSF10* | Tumor Necrosis Factor (Ligand) Superfamily, Member 10 |
|  | *VWF* | Von Willebrand Factor |
